# Telemedicine in Neurosurgical Trauma during the COVID-19 Pandemic: A Single-Center Experience

**DOI:** 10.3390/diagnostics12092061

**Published:** 2022-08-25

**Authors:** Nenad Koruga, Anamarija Soldo Koruga, Robert Rončević, Tajana Turk, Vjekoslav Kopačin, Domagoj Kretić, Tatjana Rotim, Alen Rončević

**Affiliations:** 1 Department of Neurosurgery, University Hospital Center Osijek, 31000 Osijek, Croatia; 2 Faculty of Medicine, Josip Juraj Strossmayer University of Osijek, 31000 Osijek, Croatia; 3 Department of Neurology, University Hospital Center Osijek, 31000 Osijek, Croatia; 4 Department of Diagnostic and Interventional Radiology, University Hospital Center Osijek, 31000 Osijek, Croatia

**Keywords:** telemedicine, neurosurgery, trauma, injury, COVID-19, pandemic, SARS-CoV-2

## Abstract

Telemedicine is a rapid tool that reduces the time until treatment for patients, which is especially useful for neurosurgical trauma. The aim of our study was to evaluate the use of telemedicine in neurosurgery during the COVID-19 pandemic compared with the pre-pandemic era. We assessed the utilization of telemedicine at the Department of Neurosurgery at University Hospital Center Osijek in Croatia over a timespan of one year prior to the COVID-19 pandemic and the first year of the pandemic, starting with the date of first lockdown in Croatia. For each time period, the total number of consults and specific clinical inquiries were recorded and adequately grouped as well as comprehensive patient characteristics. There were 336 consults in the pre-pandemic period and 504 in the pandemic period. The number of trauma-related consults during COVID-19 measures was significantly higher than the pre-pandemic era (288 and 138, respectively, *p* < 0.0001). Neurosurgical trauma patients requiring consults in the pandemic period were significantly older than before the pandemic (64.9 ± 18.5 and 60.6 ± 19.1, respectively, *p* = 0.03). Significantly, the number of admissions to our center and urgent surgeries did not significantly differ between these periods. Telemedicine is a cost-effective tool in the neurosurgical evaluation of patients, especially for trauma. The COVID-19 pandemic accelerated telemedicine implementation and improved neurosurgical trauma treatments.

## 1. Introduction

In recent times, digitalization has had a major impact on our society and medicine is no exception. Telemedicine is conventionally defined as the utilization of digital communications and software in order to adequately manage patients without face-to-face visits [1]. This is especially important for geographically disadvantaged patients without access to specialty care. Early skepticism towards telemedicine, primarily regarding the lack of scientific evidence for its clinical and cost effectiveness [2], did not stand the test of time. Although the initial concerns have been almost completely resolved, with time new challenges have emerged; the current spotlight is on ethical and legal issues [3]. The history of telemedicine in Croatia started in 1993 when the first teleradiology system for telepathology was organized [4]. Telehealth implementation considerably improved the quality of care, which was also evident in neurosurgical patients [5]. However, more recent data about telemedicine usage in Croatia are almost non-existent.

### 1.1. Telemedicine and COVID-19

The emergence of SARS-CoV-2 in late 2019 and the inability to control the spread of the virus worldwide required quick and effective adaptations in medicine, with the intention of reducing the burdens on healthcare workers and to more effectively contain the virus. For example, as an initial response to the pandemic, surgical specialties started cancelling elective procedures, thus increasing the capacity for potential COVID-19 patients [6]. These unique conditions permitted and necessitated the rapid implementation of remote digital technologies in healthcare. An underappreciated aspect of telemedicine is empowerment-oriented telehealth services, which are especially effective for lifestyle modifications [7]. Pandemic-induced uncertainty and fear among the general population increased stress, anxiety, and depression [8]. In this context, agent-based simulators (ABSs), as a unique patient-centered tool of e-health, promote self-care mindfulness and have shown promising results [9]. Combining ABSs with machine learning could significantly improve the wellbeing of patients without direct supervision from physicians. In addition to eliminating unnecessary face-to-face visits, the time- and cost-efficient properties of telemedicine were crucial to, and proved to be pivotal during, the COVID-19 pandemic, as evidenced by the accelerated implementation of telehealth in the last two years [10,11]. As Hollander and Carr originally speculated at the very start of the pandemic, telemedicine was ‘virtually perfect’ for these peculiar circumstances [12].

### 1.2. Telemedicine in Neurosurgery

Usual neurosurgery work involves planning for elective surgery, i.e., first-time and pre-operative visits of patients as well as planning for post-operative care and follow-ups. However, emergency surgeries are also a significant part of neurosurgical work. Telehealth can facilitate all of these cases. It should be noted that telehealth may still have a few inherent limitations regarding thorough patient examinations due to the physical absence of the physician [13]. Nonetheless, considerable progress has been made, even in the area of remote patient assessments [14,15]. Rapid assessments and treatment are especially important for neurosurgical trauma patients, where time is of the essence and can be the deciding factor in patient survival. Previous studies have shown that the majority of telemedicine neurotraumas can effectively be treated by non-neurosurgeons [16,17]. In addition, the local treatment of neurotraumas reduces needless transfers to neurosurgical departments. By using telehealth services, such patients may remain under the supervision of neurosurgeons and timely treatments can still be provided.

Public health measures such as government-enforced lockdowns, travel restrictions, and social distancing were enforced, with the main goal of reducing the spread of the virus. However, these policies also had a few major indirect consequences. In particular, several studies reported a notable decrease in the quantity of trauma patients [18,19,20], although in a few studies the patterns of injury changed somewhat. Despite the general reduction in traumas, the quantity of emergency procedures and admissions did not significantly change [18,21].

The primary aim of our study was to assess the differences in telemedicine consults in our department prior to the pandemic-related public health policies and during those policies, with a particular focus on trauma-related events. Additionally, by comparing the quantity of consults during these periods, we could assess the reliance on telemedicine in neurosurgery in Croatian healthcare before and during the pandemic as well as discuss future trends. In doing so, we could investigate the evidence for telemedicine in neurosurgery to determine its benefits, validity, and impact, especially during the period of reduced patient visits due to the global pandemic.

## 2. Materials and Methods

A retrospective chart review of telemedicine consults was performed at the Department of Neurosurgery, University Hospital Center Osijek, Croatia. The telemedicine system employed in Croatian healthcare is the Issa/Pharos system developed by VAMStec, Zagreb. Within this system, workstations are situated in smaller hospitals with radiology departments as well as in tertiary centers such as University Hospital Center Osijek. Telemedicine consults were provided between the attending neurosurgeon from our department and a referring physician from remote hospitals. As part of the telemedicine consult, the demographic information, a basic anamnesis, and an assessment of the patient were obtained by the physicians from remote hospitals and sent via the Issa application. The telemedicine workstations operated in a DICOM (digital imaging and communication in medicine) format, which enabled the transfer of neuroradiology imaging and descriptions from the remote hospital. By combining the medical imaging with elementary patient information, the attending neurosurgeon could suggest further treatment options to the referring physician. During the study period at the Department of Neurosurgery, University Hospital Center Osijek, there were 7 neurosurgeons responding to telemedicine consults. It should be emphasized that the consults received by our department were mostly related to acute pathologies.

In this study, data were collected for two time periods of an equal length (365 days): a pre-pandemic and a pandemic period. The pre-pandemic period was defined as between 11 March 2019 and 9 March 2020 whereas the pandemic period started on 11 March 2020 (the day that was defined by the Croatian government as the start of the COVID-19 epidemic in Croatia) and ended on 10 March 2021. It was the decision of the authors to have a total of 365 days in both timeframes.

**Figure 1:**
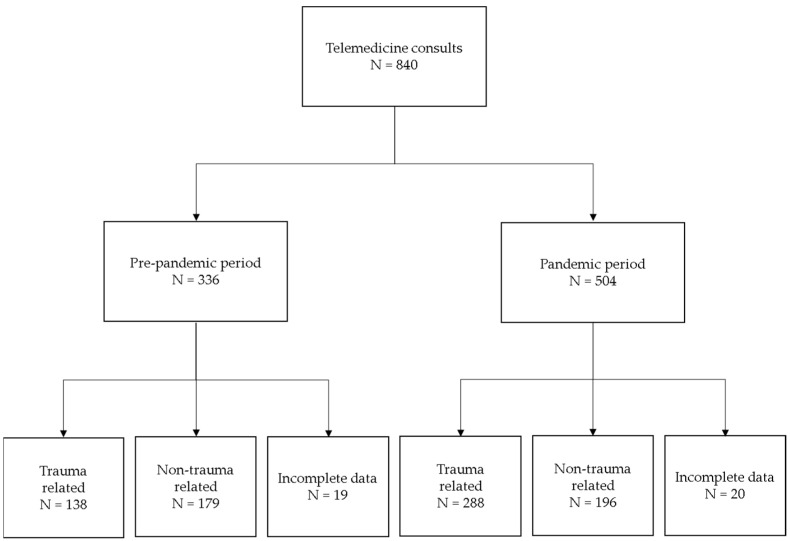
Study workflow. A retrospective chart review of telemedicine consults was performed at University Hospital Center Osijek for a total of 730 days. Consults were chronologically grouped as pre-pandemic or pandemic, as described in Materials and Methods. Depending on the etiology, consults were characterized as trauma- or non-trauma-related. Consults with unclear etiology or incomplete data were excluded from further analyses.

For every day, the number of consults was documented as well as the sex, age, and diagnosis of the patients along with the number of urgent transfers of patients to our hospital and the number of emergency surgeries. Consults missing any of these aforementioned variables were excluded from this study, Additionally, consultations were characterized as trauma-related or non-trauma-related. Consults with insufficient information about the trauma were excluded from further statistical analyses. The workflow of our study is presented in Figure 1.

### Statistics

The categorical variables were analyzed using a χ^2^ test. Comparisons of the means were analyzed by a Student’s *t*-test. The significance level was set at an α of 0.05. The analyses were performed using Microsoft Excel (Redmond, WA, USA) and Python 3.7 (Scotts Valley, CA, USA).

## 3. Results

The combined number of telemedicine inquiries during the study period was 840. A total of 336 consults were documented during the pre-pandemic period. In comparison, during the pandemic period 504 were documented (Table 1), which presented an increase of 50%. The median age of patients before the COVID-19 pandemic was 65 years (53–76) whereas the median age of patients during the pandemic was 68 years (55–79). However, the differences between the two groups did not reach a statistical significance. In the pre-pandemic period, 50 patients (14.9%) were urgently transferred to our hospital and 80% of those patients underwent emergency surgery. During the pandemic period, there were 55 urgent transfers of patients, of which 78.2% had urgent procedures. With both variables—the number of admissions and emergency surgeries—we did not detect any significant differences between the two periods.

### 3.1. Types of Consults

**Figure 2:**
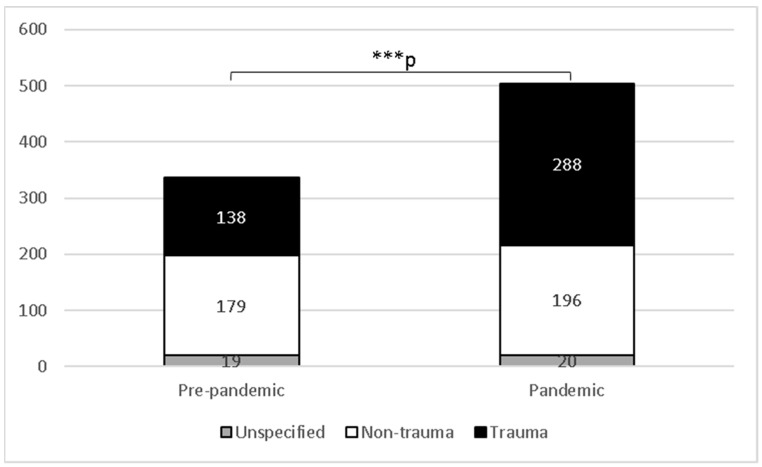
Number of telemedicine consults in pre-pandemic and pandemic periods at University Hospital Center Osijek, Croatia. *** *p* < 0.0001, χ^2^ test with Yates’s correction.

**Figure A1:**
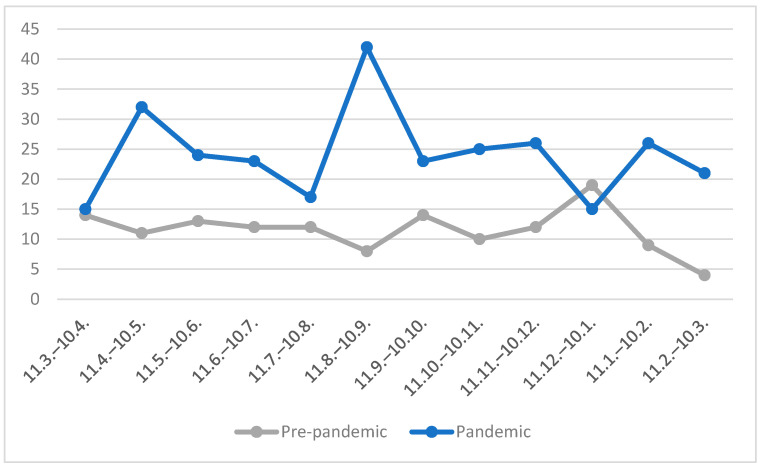
Frequency of trauma-related consults at University Hospital Center Osijek, Croatia, in pre-pandemic and pandemic periods.

In order to further analyze the telemedicine inquiries, we characterized them as either trauma-related or non-trauma-related. The results are presented in Figure 2. Of the 336 consults, 138 (41.1%) were trauma-related, 179 (53.3%) were non-trauma-related, and 19 (5.6%) were unspecified. During the pandemic period, 288 (57.1%) out of 504 consults were trauma-related, 196 (38.9%) were non-trauma-related, and 20 (4%) were unspecified. The increase in trauma-related consults after the COVID-19 outbreak was statistically significant (χ^2^ (1, *N* = 801) = 18.9, *p* < 0.0001). The trends of the trauma-related consults during the study period are presented in Figure A1, Appendix A.

### 3.2. Trauma-Related Consults

**Figure 3:**
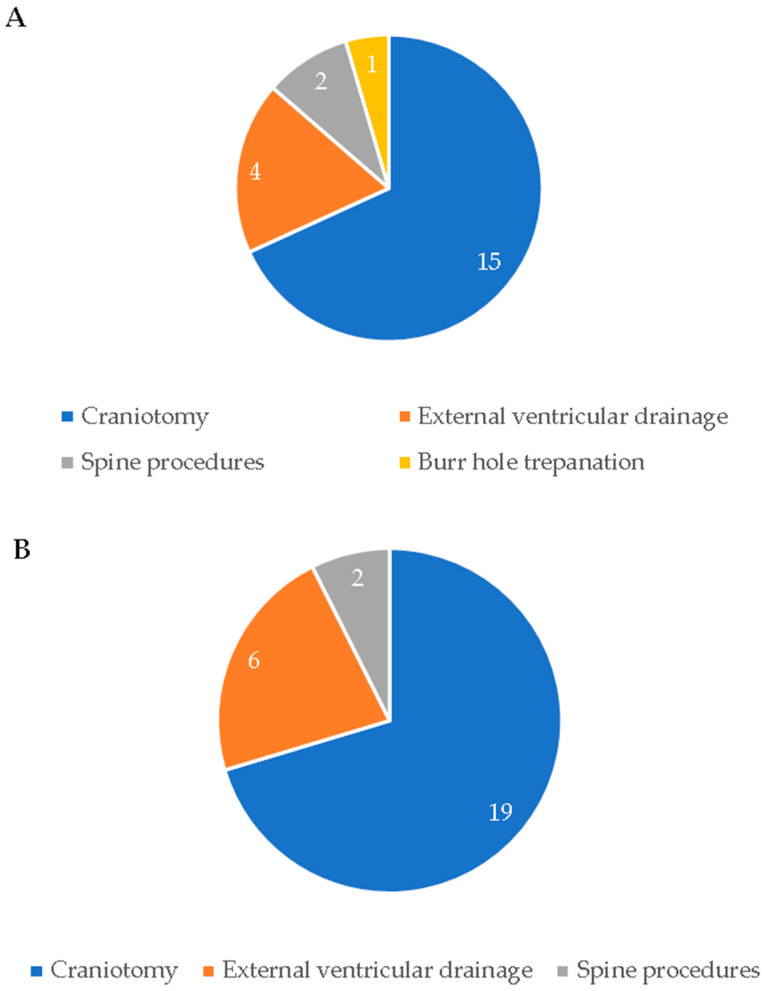
Emergency surgeries of neurosurgical trauma patients transferred to University Hospital Center Osijek in pre-pandemic (**A**) and pandemic (**B**) period.

In order to further examine the qualitative changes in the trauma-related consults, we compared the age, sex, number of urgent transfers and surgeries, and type of trauma (head or spine trauma) between the two periods. The results are presented in Table 2. The median age of the trauma patients before the COVID-19 pandemic was 64 years (48–75) whereas the median age of the trauma patients during the pandemic was 69 years (55–80). The age of the patients was significantly higher during the pandemic period compared with the pre-pandemic period (*t*(424) = 2.2, *p* = 0.03). Out of 138 pre-pandemic trauma-related inquiries, 25 (18.1%) of them required a transfer to our hospital, of which 21 (84%) underwent emergency surgery. In comparison, after the COVID-19 outbreak, out of 288 trauma-related telemedicine consults, 32 (11.1%) patients were admitted and 25 (78.1%) were urgently operated on. The types of emergency interventions are presented in Figure 3. Although the number of trauma-related consults was substantially higher during COVID-19 measures, we did not detect any statistically significant differences in the number of admissions and urgent surgeries. In our sample, the majority of trauma patients before and during the pandemic were men; 70.3% and 68.1%, respectively. The ratio of men to women regarding neurosurgical traumas did not significantly change during the study period. Head traumas were more common than spine traumas before (69.6%) as well as during the pandemic (76.6%). Notably, the head and spine trauma prevalence during these periods did not significantly differ.

## 4. Discussion

Prior to the COVID-19 pandemic, the implementation of telemedicine was faced with many obstacles such as infrastructure difficulties, problems with physical examinations, legal concerns, and a lack of data supporting efficiency [22]. Realizing the full potential of the technology was thought to be years away; the utilization of telehealth in surgery was undergoing slow, but consistent, progress [23,24]. However, with the COVID-19 outbreak, circumstances in society and medicine suddenly changed; it became necessary to implement this modality in an accelerated manner. Consequently, this reduced unnecessary hospital visits and limited the community exposure to SARS-CoV-2, thus reducing the burden on healthcare workers during the pandemic. Additionally, the rapid implementation of telemedicine improved treatments for patients in remote hospitals that did not have certain specialty care such as neurosurgeons. This was evident in our results, where the total number of telemedicine consults during the pandemic increased by 50% compared with the year prior to the COVID-19 outbreak. In other words, the average daily number of consults rose from 0.92 to 1.38. On the other hand, despite the increase in telemedicine consults, we did not observe a significant increase in admissions to our hospital or emergency surgeries. Our study provides unique insights into telemedicine trends in neurosurgery in Croatia and describes a curious increase in neurosurgical traumas during the pandemic.

### 4.1. Neurosurgical Traumas during the Pandemic in Croatia

Studies undertaken in the early phases of the stay-at-home orders reported decreasing volumes of elective neurosurgical operations [11,25,26,27]. Similarly, the quantity of neurosurgical traumas was also reported to be in a decline [28]. Furthermore, many studies reported significant decreases in general traumas, as presented in Table A1. However, a few of the studies described increases in violence-related injuries. The results presented in our study were in contrast to those findings. As previously stated, telehealth in Croatia was first organized in 1993 [4], by which a solid foundation for future improvements was laid. Our results suggested that Croatian telemedicine, particularly neurosurgical trauma, quickly and efficiently adapted to these new conditions. Overall, our department observed a considerable increase in telemedicine consults, which could be explained by rapid advancements in telemedicine technology and communication between health institutions in Croatia. Significantly, neurosurgical traumas represented a significant portion of those consults during the pandemic period. To be more precise, when compared with the period prior to COVID-19, the total number of neurosurgical traumas more than doubled (138 prior to the COVID-19 pandemic and 288 during). Although most studies reporting a decrease in traumas during the pandemic conducted their research during shorter time periods compared with ours [20,29,30,31], this does not sufficiently explain our findings. If this was the case, we would have observed less trauma in the initial few weeks after the lockdown onset, which was not the case, as presented in Figure A1. Despite an increase in general and trauma-related telemedicine consults, the emergency workload did not increase. Furthermore, the proportion of emergency trauma transfers to our hospital during the pandemic was reduced compared with the pre-pandemic era (11.1% and 18.1%, respectively), although it did not reach a statistical significance. It should also be noted that the ratio of head to spine injuries did not significantly change during the study period.

In our sample, the majority of all trauma patients, both before and during the pandemic, were men (68.8%). Previous studies also consistently described male patients as being the majority of trauma patients [32,33]. The explanation for this phenomenon is still unclear and likely multifactorial. Regarding age, the patients affected by trauma after the COVID-19 outbreak were significantly older than those before. This was also in line with findings from Scotland, which described an increase in neurosurgical traumas during COVID-19 in people over 80 years of age [34]. Public health measures might have an impact on the older population; the advised travelling restrictions and limited social exposure could have reduced the care for the elderly who were previously dependent on help from other people.

### 4.2. Future of Telemedicine in Croatia

Although the COVID-19 pandemic had many harmful consequences to society and medicine, forcing healthcare workers to quickly adapt and embrace digital technologies has paved the way for the future. According to surveys, both neurosurgical patients and neurosurgeons themselves were generally satisfied with telemedicine in the midst of the pandemic [35,36]. The inherent nature of neurosurgery being intertwined with radiographic imaging lends itself to telehealth services. Our neurosurgery department successfully adjusted in these trying circumstances during the pandemic; communication with remote hospitals was more intensive during the first year of the pandemic than before.

Non-trauma patients after telemedicine consults with a neurosurgeon were comprehensively evaluated at parent hospitals and prepared for surgery if necessary. On the other hand, the majority of trauma patients were effectively treated locally. The strategy for these patients often included follow-up consults, which are crucial for patients with a traumatic brain injury or intracranial hemorrhages [37]. Timely and precise feedback about patient neurological alterations by referring physicians in these cases is essential. If the symptoms of the patients worsened, urgent neuroradiologic imaging was usually advised, upon which another consult was conducted and, if needed, the patients were transferred and operated on.

Another less-mentioned aspect of telemedicine is in the education of residents. Even though nothing can replace the time spent in the operating room for residents, the development of virtual and augmented reality and their utilization in surgical education provides unique clinical scenarios with no real risk to patients [38]. Despite the fact that the number of elective surgeries declined during the pandemic, digitalization still enabled the continuous education of trainees despite the decreased surgical volume [39,40,41].

### 4.3. Limitations

Our study has the common problems of retrospective studies performed in a specific geographical area: a limited ability to generalize our findings, as evident by the contrasting results we observed when compared with most other studies of a similar type, and limited variables chosen by authors. In particular, we did not collect the number of follow-up telemedicine consults for trauma patients and their morbidity. Another variable we did not account for in our study was the cause of the trauma, which previous research characterized with fascinating results. University Hospital Center Osijek also faced the burden of the pandemic differently during our study period; during more critical time intervals, the intensive care unit and the hospital in general were overwhelmed. This could have led to altered results during those weeks, which we did not scrutinize further.

## 5. Conclusions

In this study, we characterized telemedicine neurosurgical trends with a focus on trauma during the first year of the pandemic in Croatia. In contrast to most of the previously published literature describing traumas during the global pandemic, the volume of neurosurgical traumas increased in eastern Croatia, which was especially pronounced in the older population. However, this increase did not have a significant impact on the total number of emergency admissions and surgeries at University Hospital Center Osijek. It would be interesting to examine telemedicine trends in Croatia over even longer timeframes and to compare them with the results from other tertiary centers in Croatia that use the Issa/Pharos system.

The COVID-19 pandemic induced fundamental shifts in modern medicine. In order to limit exposure to the virus, the accelerated implementation of digital technologies permitting swift medical decisions was crucial to not overburden healthcare workers unless absolutely necessary. Our results were an example of the telemedicine trends in Croatia; medical doctors were able to appropriately address the health problems of their patients by consulting other doctors and ensuring adequate treatments. The observed increase in telemedicine utilization in neurosurgery during the pandemic in Croatia supports the continuous implementation of e-health services in day-to-day practice. It is beneficial for patients as well as doctors to take advantage of other aspects of telehealth such as ABSs. Our department currently only uses telemedicine for communication with physicians at remote hospitals about relatively urgent cases. This is critical for neurosurgical trauma patients requiring urgent surgeries where the time until treatment must be minimized. Telemedicine practices in neurosurgery that intensified with the start of the pandemic will unequivocally extend beyond that.

## Figures and Tables

**Table 1:** Sample characteristics.

	Pre-Pandemic	Pandemic	*p*-Value
Consults	336	504	
Age	62.6 ± 17.4	64.9 ± 16.9	0.06 ^1^
Admissions	50 (14.9%)	55 (10.9%)	0.07 ^2^
Surgeries	40 (11.9%)	43 (8.5%)	0.09 ^2^

^1^ Student’s *t*-test; ^2^ χ^2^ test.

**Table 2:** Trauma-related consults during pre-pandemic and pandemic periods.

	Pre-Pandemic		Pandemic	
**Age**	60.6 ± 19.1		64.9 ± 18.5 *	
**Admissions**	25		32	
**Surgeries**	21		25	
**Type of Trauma**	Head	Spine	Head	Spine
	96	43	219	74
**Sex**	Male	Female	Male	Female
	97	41	196	92

* *p* < 0.05, Student’s *t*-test.

## Data Availability

Data are available upon request.

## Appendix A

**Table A1:** Studies evaluating trauma during COVID-19 pandemic.

Study	Key Findings
Kamine et al. [42]	Significant decrease in trauma admissions.
Park et al. [43]	Significant decrease in trauma referrals, admissions, and surgeries.
Scott et al. [44]	Significant decrease in orthopedic trauma referrals.
Ghafil et al. [45]	Transient decrease in trauma volume and then a return to baseline.
Sidpra et al. [46]	Significant increase in abusive head traumas in children.
Olding et al. [47]	Significant decrease in trauma volume, but increase in penetrating injuries.
Qasim et al. [48]	Significant decrease in trauma volume, but increase in penetrating injuries.
